# TXNDC5 Plays a Crucial Role in Regulating Endoplasmic Reticulum Activity through Different ER Stress Signaling Pathways in Hepatic Cells

**DOI:** 10.3390/ijms25137128

**Published:** 2024-06-28

**Authors:** Seyed Hesamoddin Bidooki, Cristina Barranquero, Javier Sánchez-Marco, Roberto Martínez-Beamonte, María J. Rodríguez-Yoldi, María A. Navarro, Susana C. M. Fernandes, Jesús Osada

**Affiliations:** 1 Departamento de Bioquímica y Biología Molecular y Celular, Facultad de Veterinaria, Instituto de Investigación Sanitaria de Aragón, Universidad de Zaragoza, E-50013 Zaragoza, Spain; h.bidooki94@gmail.com (S.H.B.); javiersanchezmarc@gmail.com (J.S.-M.); romartin@unizar.es (R.M.-B.); angelesn@unizar.es (M.A.N.); 2 Instituto Agroalimentario de Aragón, CITA, Universidad de Zaragoza, E-50013 Zaragoza, Spain; cbarranq@unizar.es (C.B.); mjrodyol@unizar.es (M.J.R.-Y.); 3 Institute of Analytical Sciences and Physico-Chemistry for Environment and Materials (IPREM), Universite de Pau et des Pays de l’Adour, E2S UPPA, CNRS, 64 000 Pau, France; susana.fernandes@univ-pau.fr; 4 MANTA—Marine Materials Research Group, Universite de Pau et des Pays de l’Adour, E2S UPPA, 64 600 Anglet, France; 5 Centro de Investigación Biomédica en Red de Fisiopatología de la Obesidad y Nutrición (CIBEROBN), Instituto de Salud Carlos III, E-28029 Madrid, Spain; 6 Departamento de Farmacología, Fisiología, Medicina Legal y Forense, Facultad de Veterinaria, Instituto de Investigación Sanitaria de Aragón, Universidad de Zaragoza, E-50013 Zaragoza, Spain

**Keywords:** NAFLD, liver, endoplasmic reticulum stress, TXNDC5, ATF6, EIF2AK3, PERK, ERN1, IRE1a, HSPA5, tunicamycin, palmitic acid, thapsigargin, hepatocytes

## Abstract

The pathogenesis of non-alcoholic fatty liver disease (NAFLD) is influenced by a number of variables, including endoplasmic reticulum stress (ER). Thioredoxin domain-containing 5 (TXNDC5) is a member of the protein disulfide isomerase family and acts as an endoplasmic reticulum (ER) chaperone. Nevertheless, the function of TXNDC5 in hepatocytes under ER stress remains largely uncharacterized. In order to identify the role of TXNDC5 in hepatic wild-type (WT) and TXNDC5-deficient (KO) AML12 cell lines, tunicamycin, palmitic acid, and thapsigargin were employed as stressors. Cell viability, mRNA, protein levels, and mRNA splicing were then assayed. The protein expression results of prominent ER stress markers indicated that the ERN1 and EIF2AK3 proteins were downregulated, while the HSPA5 protein was upregulated. Furthermore, the ATF6 protein demonstrated no significant alterations in the absence of TXNDC5 at the protein level. The knockout of TXNDC5 has been demonstrated to increase cellular ROS production and its activity is required to maintain normal mitochondrial function during tunicamycin-induced ER stress. Tunicamycin has been observed to disrupt the protein levels of HSPA5, ERN1, and EIF2AK3 in TXNDC5-deficient cells. However, palmitic acid has been observed to disrupt the protein levels of ATF6, HSPA5, and EIF2AK3. In conclusion, TXNDC5 can selectively activate distinct ER stress pathways via HSPA5, contingent on the origin of ER stress. Conversely, the absence of TXNDC5 can disrupt the EIF2AK3 cascade.

## 1. Introduction

Non-alcoholic fatty liver disease (NAFLD) is highly prevalent in the general population [1] and is now the most common liver pathology in Western countries, posing a significant public health concern [2]. NAFLD develops naturally without alcohol abuse, but its precise pathophysiology is still unknown [3,4]. The pathogenic conditions associated with NAFLD range from simple steatosis to steatohepatitis (NASH) and cirrhosis, which can ultimately result in hepatocellular cancer [5,6]. According to the ‘two hits’ hypothesis, fat accumulation is the initial step. This accumulation makes the liver more susceptible to the damaging effects of one or more additional factors, leading to the development of steatohepatitis and fibrosis [7]. Multiple factors are believed to contribute to the pathogenesis of NAFLD, including hepatic lipid buildup, insulin resistance, oxidative stress, apoptotic pathways, and adipocytokine production [8].

The endoplasmic reticulum (ER) is the primary organelle for secretory pathways in all eukaryotic cells [9,10]. The ER serves as the entry point into the secretory system and plays a crucial role in maintaining cellular calcium homeostasis, lipid production, and transmembrane protein folding. Therefore, preserving ER homeostasis is a crucial element of cellular physiology [11]. ER processes can be impaired by various factors, leading to the accumulation of unfolded proteins and activation of the unfolded protein response (UPR). The UPR is responsible for restoring ER balance and promoting survival [12,13,14]. ER stress has been associated with various disorders, such as cardiovascular, endocrine, and nervous system disorders [15,16]. Under sustained and severe ER stress, the UPR can become cytotoxic, leading to apoptosis, instead of being cytoprotective. The UPR signaling pathways involve three distinctive signaling transduction mechanisms: activating transcription factor 6 (ATF6), eukaryotic translation initiation factor 2 alpha kinase 3 (EIF2AK3, also known as protein kinase RNA-like ER kinase, PERK), and endoplasmic reticulum to nucleus signaling 1 (ERN1) [15]. ATF6 is a type II transmembrane protein that serves as an ER stress sensor. It activates chaperones and elements of the endoplasmic reticulum-associated protein degradation (ERAD) pathway [17]. Furthermore, it can activate the target genes DNA-damage inducible transcript 3 (DDIT3), glucose-regulated protein 94 (GRP94), and heat shock protein family A member 5 (HSPA5)/BIP [18]. EIF2AK3 reduces the workload of misfolded proteins by inhibiting mRNA translation during ER stress and limiting further synthesis [19]. To translate activating transcription factor 4 (ATF4), one of the UPR-dependent signaling proteins, EIF2AK3, phosphorylates eukaryotic initiation factor 2 (EIF2α). This process also activates DDIT3, which leads to the production of reactive oxygen species [20,21]. The transmembrane protein ERN1 regulates its own expression and functions as an endoribonuclease and protein kinase. It produces a potent transcription activator called X-box binding protein 1 (XBP1). ERN1 is related to different translocon proteins such as signal sequence receptor, beta (SSR2) and SEC61 translocon subunit alpha 1 (SEC61A1) [22], which may improve the ER’s ability to fold proteins [23,24,25]. HSPA5 detects these proteins and plays a crucial role in managing accumulated proteins [26].

As mentioned, the ER is the site where nascent peptides are correctly folded. A number of redox proteins, including members of the protein disulfide isomerase (PDI) family, interact to coordinate this complex process. TXNDC5, an essential member of the PDI family, participates in a series of disulfide bond exchange events that converge in folding newly synthesized polypeptides into their mature form [27,28,29]. TXNDC5 protects liver cells from stress-induced apoptosis and is essential for signal transduction and cancer development [30,31]. The aim of this research was to investigate how the TXNDC5 protein interacts in the context of ER stress. Specifically, we aim to examine its role through the three key signaling pathways involved in ER stress: ATF6, EIF2AK3, and ERN1. To this end, mouse hepatocytes with and without TXNDC5 will be exposed to known inducers of ER stress, and the key molecular mechanisms will be investigated. This experimental setup will provide a comprehensive understanding of the functional significance of TXNDC5 in maintaining cellular homeostasis under ER stress conditions.

## 2. Results

### 2.1. TXNDC5 Deletion Alters ER Stress-Related Expressions

TXNDC5 was completely knocked out in AML12 cells (KO), as evidenced by significantly decreased mRNA and protein expression levels compared to wild-type AML12 cells (WT) (Supplementary Figure S1). To investigate the role of TXNDC5 in regulating ER stress markers in AML12 cells, RT-qPCR was performed to assess the mRNA expression of *Atf6*, *Ern1*, *Xbp1*, *Eif2ak3*, *Atf4*, and *Ddit3*; as primary genes involved in the three different ER stress cascades, *Ssr2* and *Sec61a1*; as translocon genes and *Hspa5*; as an ER protein recognition chaperon in WT and TXNDC5-KO AML12 cells. Table 1 shows that the knockout of TXNDC5 in AML12 cells resulted in downregulation of ER genes *Ern1*, *Eif2ak3*, *Xbp1*, and *Sec61α1* compared to WT cells. Additionally, TXNDC5 inactivation significantly increased the mRNA levels of *Hspa5*, *Atf4*, and *Ddit3*. However, the absence of TXNDC5 did not cause any significant change in the mRNA levels of *Atf6* and *Ssr2*. To confirm these results at the protein level, the most prominent ER stress markers were selected for Western blot analysis. The results indicated that the ERN1 and EIF2AK3 proteins were downregulated, while the HSPA5 protein was upregulated. However, the ATF6 protein did not exhibit any significant changes in the absence of TXNDC5 at the protein level. These results are fully compatible with the mRNA expression outcomes and suggest that TXNDC5 plays a critical role in maintaining the stability of ER genes in mouse hepatocytes.

### 2.2. Effect of ER Stressors (Tunicamycin, Palmitic Acid, or Thapsigargin) on the Survival Rate of AML12 Cell Lines

**Figure 1:**
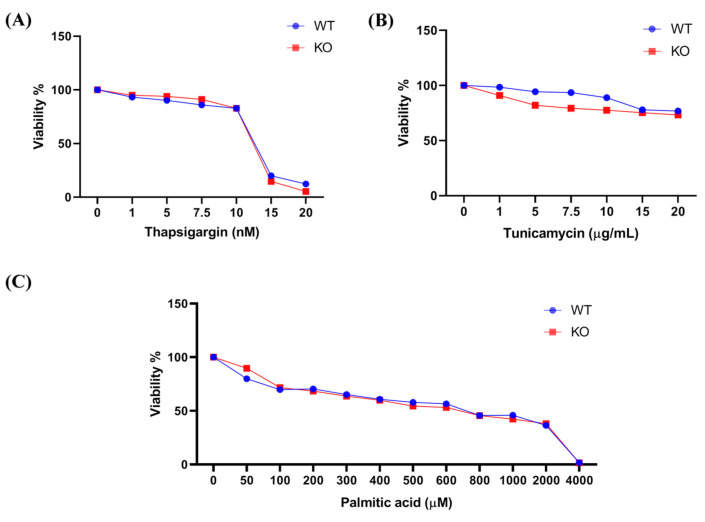
The cell viability evaluation of mouse hepatocytes upon ER stress. After 24 h, treated with different concentrations of thapsigargin, tunicamycin, and palmitic acid, TXNDC5 absence did not produce a substantial difference in cell viability of WT compared to KO cells. Viability rates were indicated in the exposure of (**A**) thapsigargin, (**B**) tunicamycin, and (**C**) palmitic acid. WT: normal mouse hepatocyte AML12 cells, KO: TXNDC5-deficient AML12 cells.

Both WT and TXNDC5-KO cells were incubated in the presence of pharmacological inducers of the UPR and ER stress: thapsigargin, an inhibitor of the ER calcium pump, tunicamycin, an inhibitor of N-linked glycosylation, and palmitic acid, an inhibitor of thapsigargin-sensitive calcium stores and a representative saturated fatty acid [22,32]. Figure 1 shows the cell viability of WT and TXNDC5-KO cells. After 24 h of exposure to different stressor concentrations, the cell viability of TXNDC5-KO cells was found to be the same as that of WT cells. Based on these results, we selected 12.5 nM of thapsigargin as an inducer of severe stress, with a viability rate of approximately 40% (Figure 1A). We also selected 1 and 20 µg/mL of tunicamycin as conditions of slight and moderate stress, respectively, with viability rates of 90% and 75%, respectively (Figure 1B). Additionally, 600 µM palmitic acid was selected as a representative of substantial stress, with a viability rate of 55% (Figure 1C), to assess the expression patterns of ER stress genes.

### 2.3. The Knockout of TXNDC5 Increases Reactive Oxygen Species in Hepatic Cells

**Figure 2:**
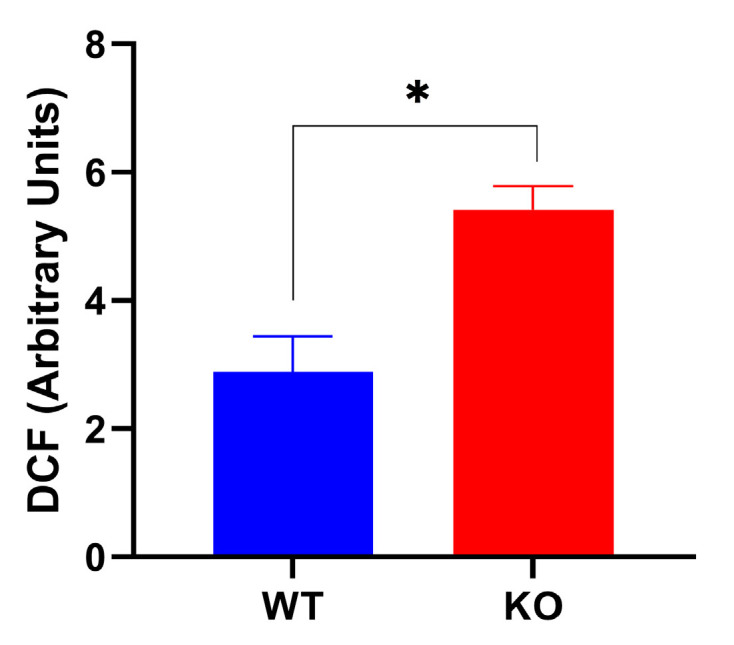
The impact of TXNDC5 deficiency on ROS production. WT: normal mouse hepatocyte AML12 cells, KO: TXNDC5-deficient AML12 cells. Statistical analyses were conducted according to Mann–Whitney’s U-test; * *p* < 0.05.

The intracellular ROS levels were determined by measuring the dichlorodihydrofluorescein (DCF) production in WT and TXNDC5-KO-AML12 cells. The results, as shown in Figure 2, indicate that TXNDC5-KO cells exhibited a significant increase in ROS production compared to WT cells. This suggests that TXNDC5 regulates ROS production in AML12 cells and its absence leads to an increase in ROS levels. Furthermore, to determine whether ER stressors can induce oxidative stress, WT and TXNDC5-KO cells were incubated with different concentrations of thapsigargin, palmitic acid, and tunicamycin for 24 h. However, no significant increase in intracellular ROS was detected in either group (Figure S2). These results suggest that the concentration used for inducing ER stress did not enhance further ROS production and indicate that these ER stressors may affect different pathways than oxidative stress.

### 2.4. TXNDC5 Activity Is Required to Maintain Normal Mitochondrial Function during Tunicamycin-Induced ER Stress

**Figure 3:**
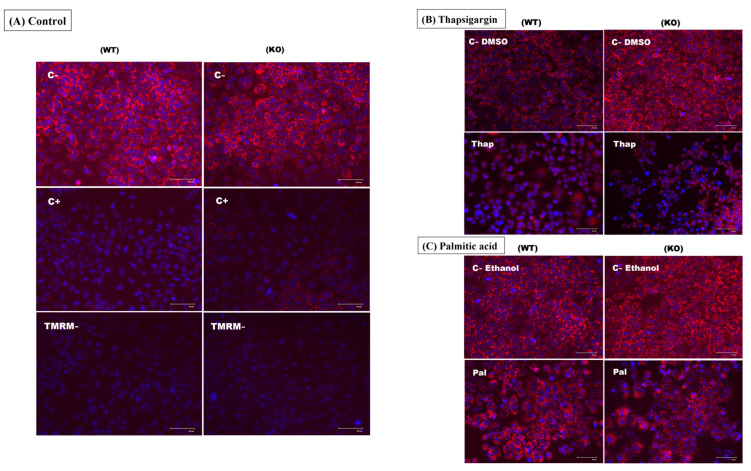

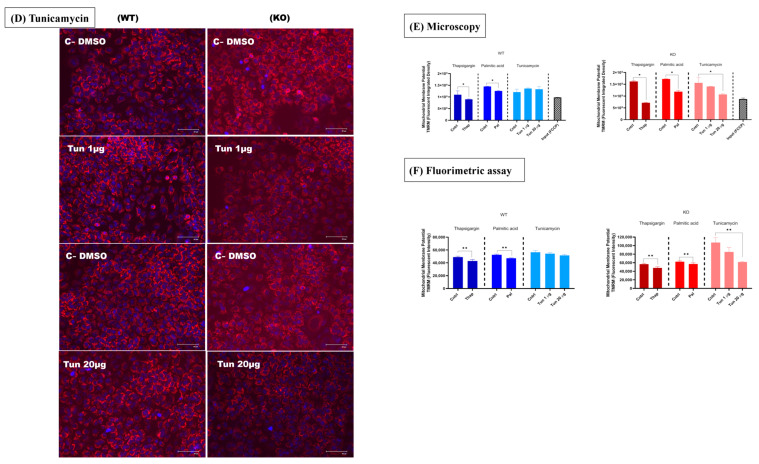
The mitochondrial membrane potential was evaluated using the TMRM assay in the following conditions: (**A**) Control negative (C−) refers to cells that were not subjected to any treatment. Control positive (C+) refers to cells that were treated with carbonylcyanide 4-(trifluoromethoxy)phenylhydrazone (FCCP), an uncoupler that will reduce the mitochondrial membrane potential and prevent staining by TMRM. TMRM negative (TMRM−) refers to cells that were not stained by TMRM. (**B**) (C− DMSO) refers to the cells that were treated with DMSO as a control negative and (Thap) refers to the cells that were treated with 6.25 nM thapsigargin. (**C**) (C− Ethanol) refers to the cells that were treated with ethanol as a control negative and (Pal) refers to the cells that were treated with 300 µM palmitic acid. (**D**) (C− DMSO) refers to the cells that were treated with DMSO as a control negative, (Tun 1 µg) refers to the cells that were treated with 1 µg/mL tunicamycin, and (Tun 20 µg) refers to the cells that were treated with 20 µg/mL tunicamycin. (**A**–**D**) The red-fluorescent probe (TMRM) localizes in mitochondria and detects mitochondrial membrane depolarization. The blue-fluorescent probe (NucBlue) detects the cellular nucleus. (**E**) The fluorescent integrated density of the red probe in the aforementioned conditions was evaluated through fluorescent microscopy, with the resulting data subsequently analyzed using the ImageJ (Fiji) software version windows 64-bit Java 8. (**F**) The fluorescent intensity of TMRM was assessed using a microplate fluorimeter at excitation/emission of 548/575 nm to corroborate the microscopic visualization results. WT: normal mouse hepatocyte AML12 cells, KO: TXNDC5-deficient AML12 cells. The statistical analyses were conducted in accordance with the Mann–Whitney U-test, with the following significance levels: * *p* < 0.05, ** *p* < 0.01.

As the canonical function of TXNDC5 is to assist protein folding in the ER, we investigated whether depletion of TXNDC5 or induction of ER stress could potentially damage mitochondria. Therefore, we used the loss of mitochondrial membrane potential (MMP) as a biomarker of effect. The tetramethylrhodamine, methyl ester (TMRM) assay was utilized to evaluate the loss of MMP in hepatic cells either by fluorimetric or microscopic procedures. The knockout of TXNDC5 did not significantly affect the mitochondrial function of the cells, as evidenced by the lack of decrease in MMP values (Figure 3A). Additionally, the results indicate a different response to ER stressors. Whereas 6.25 nM thapsigargin (Figure 3B) or 300 µM palmitic acid (Figure 3C) caused a decrease in MMP values, this was not the case for tunicamycin at the tested concentrations of 1 and 20 μg/mL (Figure 3D)) in AML12 WT cells after 24 h. In contrast, TXNDC5-KO cells exhibited altered MMP levels when exposed to tunicamycin at those concentrations, like the other ER stressors, after 24 h of exposure. The results of both procedures in WT cells indicate that tunicamycin did not decrease MMP levels (Figure 3E,F). However, in the absence of TXNDC5, tunicamycin at 20 µg/mL significantly reduced MMP levels. The lack of effect in WT cells may be due to tunicamycin’s non-interaction with the mitochondria of AML12 cells. However, in TXNDC5-KO cells, the absence of TXNDC5 may cause damage to the mitochondrial membrane when exposed to tunicamycin. These results suggest that TXNDC5 activity is necessary to maintain normal mitochondrial function during tunicamycin–ER stress.

### 2.5. The Upregulation of TXNDC5 in Hepatic Cells Is Dependent on Increased ER Stress Induced by Thapsigargin, Palmitic Acid, and Tunicamycin

**Figure 4:**
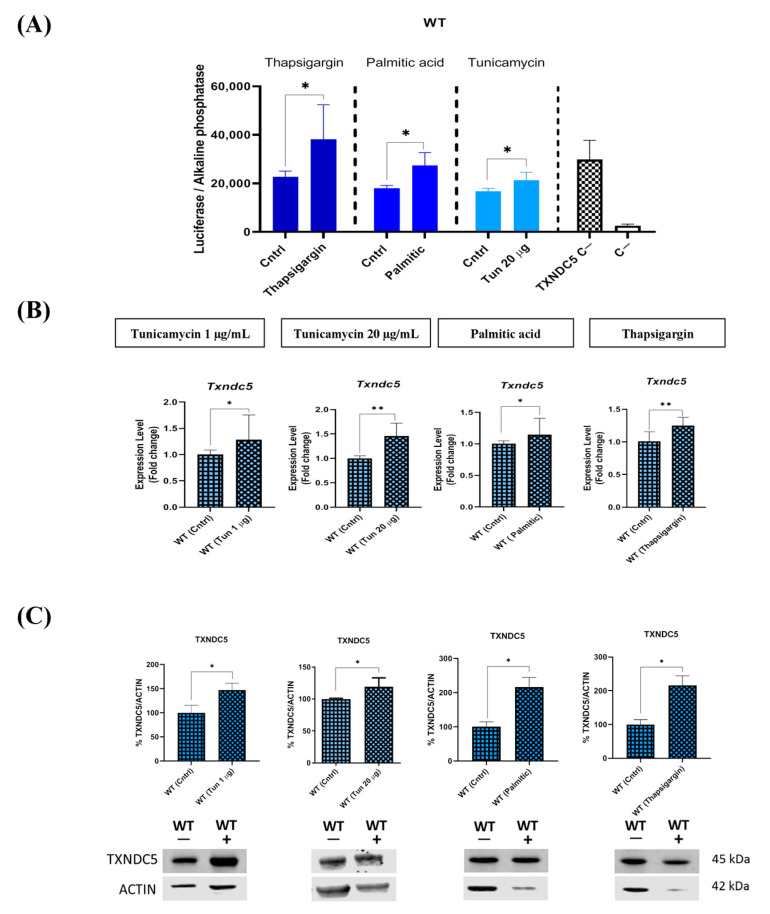
Transcriptional, mRNA, and protein responses of TXNDC5 to different stressors. (**A**) The influence of tunicamycin, palmitic acid, or thapsigargin stressors on the TXNDC5 promoter. The TXNDC5 C− and C− labels represent cells that have been transfected or cells that have not been transfected with the TXNDC5 promoter, respectively, in the absence of stressors. (**B**) mRNA levels of *Txndc5* and (**C**) protein level of TXNDC5 in WT and TXNDC5-KO cells exposed to 1 µg/mL (first column), or 20 µg/mL of tunicamycin (second column), 600 µM palmitic acid (third column), and 12.5 nM thapsigargin (fourth column). Significant augments were observed in TXNDC5 protein and mRNA expression for all stressors. WT: normal mouse hepatocyte AML12 cells, KO: TXNDC5-deficient AML12 cells. The statistical tests were conducted using the Mann–Whitney U-test. The significance levels were: * *p* < 0.05, ** *p* < 0.01.

To investigate whether ER stressors regulate the TXNDC5 promoter through distinct cascades, we transfected mouse TXNDC5 promoter–luciferase constructs into AML12 hepatic cells. AML12 WT cells were treated with thapsigargin, palmitic acid, and tunicamycin, and TXNDC5 promoter–luciferase ratios were quantified. The experiments demonstrated that the TXNDC5 promoter was induced by all stressors (Figure 4A). These data indicate that the upregulation of TXNDC5 in hepatic cells is dependent on increased ER stress induced by thapsigargin, palmitic acid, or tunicamycin. To confirm the results of the TXNDC5 promoter, assays were conducted to quantify Txndc5 mRNA expression and protein level under different stressors. In accordance with the profibrotic effects of TXNDC5 signaling previously described [27], tested stressors were observed to increase the expression and protein level of TXNDC5 in WT cells (Figure 4B,C).

### 2.6. Absence of TXNDC5 Abolishes the Induction of ATF6 and HSPA5 Expressions following Palmitic Acid Incubation

**Figure 5:**
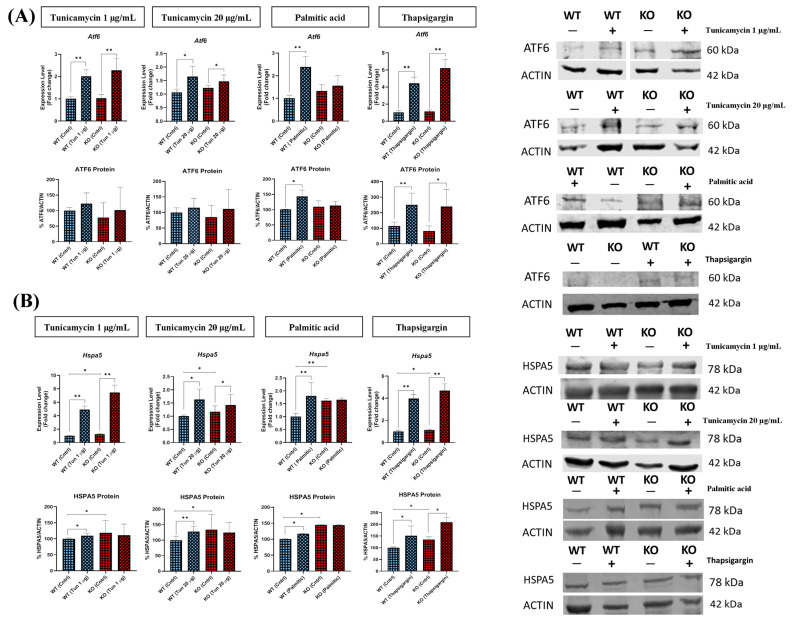
The mRNA and protein levels of (**A**) ATF6 and (**B**) HSPA5 in WT and TXNDC5-KO cells exposed to 1 (first column) or 20 µg/mL (second column) tunicamycin, 600 µM palmitic acid (third column), and 12.5 nM thapsigargin (fourth column). A notable induction was observed for ATF6 and HSPA5 mRNA and protein levels in WT cells under ER stress conditions. In the presence of palmitic acid, ATF6 levels remained unaltered in TXNDC5-KO cells. TXNDC5-KO cells demonstrated no alterations in HSPA5 expression in the presence of palmitic acid and tunicamycin. WT: normal mouse hepatocyte AML12 cells, KO: TXNDC5-deficient AML12 cells. Statistical analyses were conducted according to Mann–Whitney’s U-test; * *p* < 0.05, ** *p* < 0.01.

As illustrated in Figure 5A, the mRNA expression of *Atf6* was elevated in a variety of ER stress conditions in WT cells. The elimination of TXNDC5 in cells resulted in the abolition of the induction of *Atf6* by palmitic acid, while the changes observed in the presence of tunicamycin or thapsigargin remained unaffected. These results were corroborated at the protein level, although no significant difference was observed in ATF6 protein expression in WT and TXNDC5-KO cells following tunicamycin exposure. These results demonstrate that tunicamycin can alter the expression of ATF6 at the mRNA level, but not at the protein level. A similar pattern was observed in the case of *Hspa5* mRNA levels (Figure 5B). The deletion of TXNDC5 in cells resulted in the disruption of the induction of *Hspa5* by palmitic acid, while the changes observed in the presence of tunicamycin or thapsigargin remained unaffected. However, the HSPA5 protein level revealed that the absence of TXNDC5 abolished the HSPA5 expression induced by tunicamycin and palmitic acid. Consequently, TXNDC5 plays a pivotal role in the regulation of ATF6 and HSPA5 expression in response to palmitic acid-induced ER stress.

### 2.7. EIF2AK3 Cascade Is Disrupted in TXNDC5 Knockout Cells Exposed to Tunicamycin and Palmitic Acid

**Figure 6:**
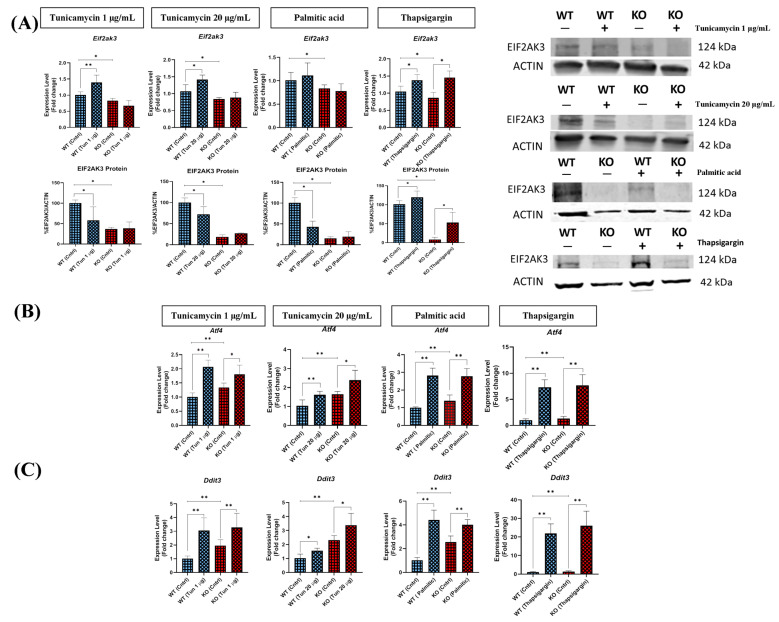
Expression level of (**A**) EIF2AK3, (**B**) *Atf4*, and (**C**) *Ddit3* in WT and TXNDC5-KO cells subjected to the 1 µg/mL tunicamycin (first column), 20 µg/mL tunicamycin (second column), palmitic acid (600 µM) (third column), and thapsigargin (12.5 nM) (fourth column). All WT cells exhibited *Eif2ak3* expression changes, whereas TXNDC5-KO cells demonstrated no alterations in ER stress, with the exception of thapsigargin. The EIF2AK3 protein levels were downregulated in WT cells in the presence of tunicamycin and palmitic acid. However, this downregulation was not observed in TXNDC5-KO cells. The mRNA levels of *Atf4* and *Ddit3* in WT and TXNDC5-KO cells exhibited a significant induction under conditions of ER stress. WT: normal mouse hepatocyte AML12 cells, KO: TXNDC5-deficient AML12 cells. Mann–Whitney’s U-test was used for statistical analysis; * *p* < 0.05, ** *p* < 0.01.

To ascertain the impact of TXNDC5 depletion on the EIF2AK3 cascade, previously designated as PERK, the mRNA and protein expression levels of EIF2AK3 were quantified. Following incubation with the three stressors, elevated *Eif2ak3* expressions were observed in WT cells (Figure 6A). However, the protein levels of EIF2AK3 showed a different pattern and displayed decreases following tunicamycin and palmitic acid, respectively. Conversely, the absence of TXNDC5 abolished the observed changes. Of note, thapsigargin induced the EIF2AK3 in both WT and TXNDC5-KO cells despite the profound decrease in EIF2AK3 expression in TXNDC5-KO cells (Figure 6A).

To evaluate the effect of EIF2AK3 disruption, the mRNA expression of other members of the EIF2AK3 cascade, including *Atf4* (Figure 6B) and *Ddit3* (Figure 6C), was assessed. There were significant increases in the mRNA levels of these genes in both WT and TXNDC5-KO cells exposed to ER stressors. Furthermore, the absence of TXNDC5 did not result in a decrement in mRNA levels of *Atf4* and *Ddit3* in TXNDC5-KO cells compared to WT cells. Consequently, TXNDC5 plays a prominent role in the stability of the EIF2AK3 cascade in tunicamycin and palmitic acid conditions, although it did not affect the *Atf4* and *Ddit3* mRNA expressions in hepatic cells.

### 2.8. TXNDC5 Deficiency Alters the ERN1 Pathway in Tunicamycin ER Stress

**Figure 7:**
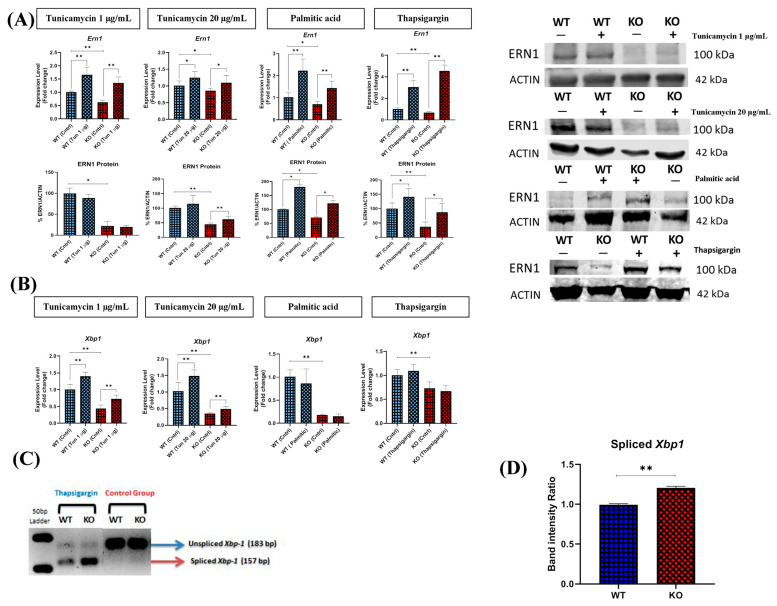
(**A**) Protein and mRNA levels of ERN1 and (**B**) *Xbp1* transcripts in WT and TXNDC5-KO cells exposed to the 1 µg/mL tunicamycin (first column), 20 µg/mL tunicamycin (second column), palmitic acid (600 µM) (third column), and thapsigargin (12.5 nM) (fourth column). A notable elevation in the level of *Ern1* mRNA was observed in both TXNDC5-KO and WT cells in response to all stressors. However, WT cells exhibited no discernible increase in the level of ERN1 protein compared to TXNDC5-KO cells in the presence of tunicamycin. In both WT and TXNDC5-KO cells, there were no changes in *Xbp1* mRNA levels in response to palmitic acid or thapsigargin. (**C**) Cells revealed a substantial spliced form of *Xbp1* mRNA in stress induced by thapsigargin. (**D**) The band intensity ratio of TXNDC5-KO cells showed significant induction of spliced form of *Xbp1* under ER stress conditions compared to the WT cells. WT: normal mouse hepatocyte AML12 cells, KO: TXNDC5-deficient AML12 cells. Statistical analysis was carried out by Mann–Whitney U-test; * *p* < 0.05, ** *p* < 0.01.

To evaluate the effects of TXNDC5 on the ERN1 cascade, previously known as IRE1, the mRNA levels of key members of this ER pathway were examined. In fact, *Ern1* exhibited elevated mRNA levels in WT and TXNDC5-KO hepatic cells following treatment with various ER stress inducers (Figure 7A). Nevertheless, this increase was not observed in WT cells in the tunicamycin condition at the protein level. The data indicated that TXNDC5 deficiency resulted in changes in ERN1 protein levels in tunicamycin stress and the ERN1 protein expression was not affected by the absence of TXNDC5 in the context of palmitic and thapsigargin stress. To elucidate the role of XBP1 in this pathway in AML12 cells, as shown in Figure 7B, *Xbp1* expression was significantly induced in tunicamycin ER stress circumstances in both WT and TXNDC5-KO cells. However, there was not a significant induction in thapsigargin and palmitic conditions. These findings demonstrate that the absence of TXNDC5 does not alter the expression of *Xbp1*, and that palmitic acid and thapsigargin are unable to induce the mRNA expression of *Xbp1*. To directly observe the activation of the ERN1–XBP1 pathway in mouse hepatic cells, estimation of *Xbp1* mRNA splicing intensity was employed. This analysis revealed that *Xbp1* mRNA splicing was significantly elevated in the ER stress induced by thapsigargin compared to the control group (Figure 7C). In accordance with the level of *Xbp1* mRNA splicing observed in the thapsigargin condition, TXNDC5-KO cells exhibited a higher spliced form than WT cells (Figure 7D). In conclusion, these findings indicate that the ERN1–XBP1 pathway is activated under conditions of thapsigargin ER stress in this hepatic cell line, and that the absence of TXNDC5 can induce the *Xbp1* spliced form. Moreover, the upregulation of ERN1 may alter the splicing pattern of *Xbp1*, rather than merely influencing its mRNA expression.

### 2.9. Expression of Ssr2 and Sec61α1 ER Protein-Translocon Channels Are Selectively Influenced by the Absence of TXNDC5

**Figure 8:**
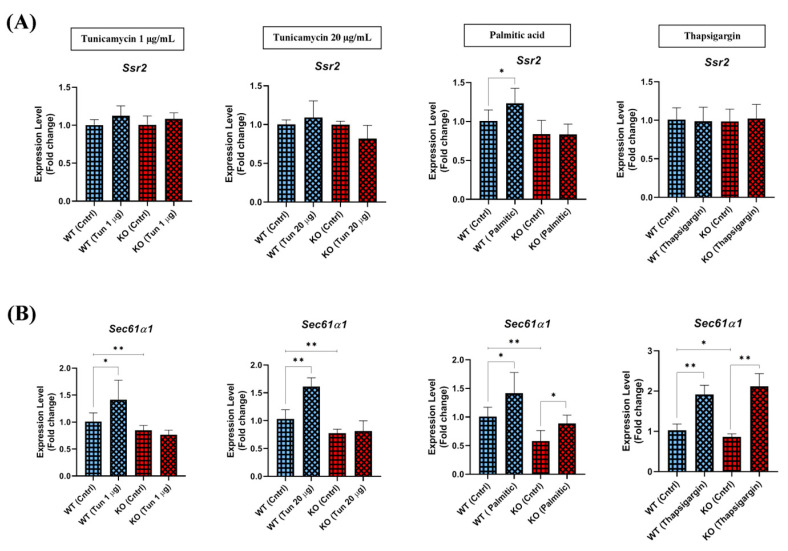
The mRNA levels of the (**A**) *Ssr2* and (**B**) *Sec61α1* genes were quantified in WT and TXNDC5-KO cells exposed to the following treatments: 1 µg/mL tunicamycin, 20 µg/mL tunicamycin, 600 µM palmitic acid, and 12.5 nM thapsigargin. The expression level of *Ssr2* was found to be increased only in WT cells exposed to palmitic acid. The expression of *Sec61α1* was elevated in WT and TXNDC5-KO cells subjected to palmitic acid and thapsigargin stress, and there was a disruption in TXNDC5-KO cells relative to WT cells in the presence of tunicamycin. WT: normal mouse hepatocyte AML12 cells, KO: TXNDC5-deficient AML12 cells. Mann–Whitney U-test was used for statistical analysis; * *p* < 0.05, ** *p* < 0.01 were considered significant.

A significant proportion of the most robust and selective ERN1 transcriptional targets identified in the UPR experiments were ER protein-translocon channels or their associated proteins [22]. As illustrated in Figure 8A, all stressors tested failed to induce the *Ssr2* gene in WT and TXNDC5-KO cells, with the exception of palmitic acid, which induced *Ssr2* mRNA expression in WT cells. This result indicates that the absence of TXNDC5 can impede the activation of *Ssr2* in response to palmitic acid exposure. However, when *Sec61α1* mRNA expression was assayed, it was observed that the different ER stressors induced its expression in WT and TXNDC5-KO cells, with the exception of the tunicamycin condition. In the presence of tunicamycin (Figure 8B), the induction of this gene was particularly dependent on TXNDC5, such that *Sec61α1* mRNA levels could not be induced in TXNDC5-KO hepatic cells. The outcomes demonstrated that the expression of *Ssr2* and *Sec61α1* ER protein-translocon channels is selectively influenced by the absence of TXNDC5 depending on the used stressor.

## 3. Discussion

The objective of this work was to investigate the influence of TXNDC5 on gene and protein expressions associated with ER stress pathways. To this end, a hepatic cell line lacking TXNDC5 was generated. The results demonstrated that TXNDC5 deficiency resulted in reduced *Ern1*, *Xbp1*, *Eif2ak3*, and *Sec61a1* expression, increased *Hspa5*, *Atf4*, and *Ddit3* expression, and did not change *Atf6* and *Ssr2* expressions. At the protein level, the absence of TXNDC5 was associated with a reduction in ERN1 and EIF2AK3 expression, while the HSPA5 protein was upregulated. Nevertheless, the ATF6 protein did not exhibit any change. The absence of TXNDC5 did not alter the pattern of mortality induced by the three known ER stressors, tunicamycin, palmitic acid, and thapsigargin, in the AML12 cell line, despite the observed enhancement of cellular ROS levels in TXNDC5-deficient cells. Nevertheless, it is evident that TXNDC5 is solely responsible for the maintenance of mitochondrial ROS levels during tunicamycin-induced ER stress. The use of these three agents resulted in elevated *Atf6*, *Hspa5*, *Atf4*, *Ddit3*, *Ern1*, and *Sec61a1* expressions in WT AML12 cells, with the exception of a lack of induction of *Eif2ak3*, *Xbp1*, and *Ssr2* in the presence of palmitic acid, thapsigargin, and tunicamycin, respectively.

A more complex outcome was observed in TXNDC5-deficient cells. In this context, while ATF6, HSPA5, and EIF2AK3 demonstrated no induction of expression in the presence of palmitic acid and tunicamycin, induction was observed in ERN1. *Xbp1* and *Ssr2* exhibited similar expression patterns to those observed in WT cells. However, in the presence of thapsigargin, the absence of TXNDC5 facilitated the splicing of *Xbp1*. Nevertheless, the expression of *Sec61a1* was disrupted by the lack of TXNDC5 in the presence of tunicamycin. Overall, TXNDC5 may regulate ER activity and is particularly involved in the palmitic acid-induced response of ATF6 and HSPA5 genes, as well as an attenuated response of EIF2AK3 signaling.

TXNDC5 has been linked to a multitude of cellular processes as an ER molecular chaperone, as evidenced by previous research [29]. As illustrated in Table 1, TXNDC5 deficiency was associated with reduced expression of ERN1, EIF2AK3, *Xbp1*, and *Sec61a1*, while expressions of HSPA5, *Atf4*, and *Ddit3* were elevated. Other studies have also demonstrated a correlation between TXNDC5 and these proteins. In this context, the fragmentation of the ER and the altered expression of numerous ER proteins, including ATF6, HSPA5, ATF4, and CCAAT/enhancer-binding protein homologous protein, are caused by the downregulation of TXNDC5 in pancreatic β-cells [33,34,35]. In addition, low TXNDC5 levels have been observed to result in increased expression levels of HSPA5, *Ddit3*, and *eIF2a*, which collectively lead to ER stress and an increase in misfolded proteins in pancreatic β-cells [34]. In summary, TXNDC5 can activate ER stress cascades via HSPA5, either directly or indirectly.

In this report, three stressors have been utilized: tunicamycin, an inhibitor of GlcNAc phosphotransferase, causes a significant accumulation of unfolded proteins, activates the UPR, increases *Txndc5*, and ultimately results in cell death by apoptosis in human head-and-neck carcinoma cells [36]. Palmitic acid is a saturated fatty acid that causes ER stress and increases the UPR in well-differentiated hepatocyte cell lines [37,38]. Thapsigargin, an inhibitor of Ca^2+^ ATPase, alters the concentration of Ca2+ within the ER lumen of mouse hepatocytes [39]. In the current study, all of the agents induced dose-dependent mortality in AML12 cells (Figure 1) and promoted TXNDC5 expression (Figure 4). It has been demonstrated in various studies that *Txndc5* is overexpressed in laryngeal squamous carcinoma cells, colorectal cancer cells, and liver and kidney cells under circumstances of ER stress [40,41,42]. In light of these observations, it can be postulated that TXNDC5 plays a role in the ER stress response induced by these agents and may act as a protective factor. However, the absence of TXNDC5 did not result in any alterations to the survival patterns, in stark contrast with other agents such as H_2_O_2_ [28]. Consequently, the absence of TXNDC5 is insufficient to alter cell survival in response to these ER stressors.

Our findings indicate that the deficiency of TXNDC5 can markedly induce cellular ROS in hepatocytes (Figure 2). These data corroborate the previous results demonstrating the enhancement of ROS in the absence of TXNDC5 in endometrial, ovarian, and colorectal tissues. In endometrial cancer cells where NR4A1 is silenced, the major source of ROS is associated with the downregulation of TXNDC5 and IDH1. This is supported by a significant increase in ROS and oxidative/ER stress after the silencing of TXNDC5 [43]. The ER has dynamic membrane contact with mitochondria, which are referred to as mitochondria–ER contacts. This contact plays an important role in regulating mitochondrial function. For instance, calcium transfer at these sites could cause calcium overload in mitochondria and initiate apoptosis. The malfunction of mitochondria and the disruption of protein translocation, translation, and folding within this organelle may result in the phenomenon of “mitochondrial stress”. ER stress and mitochondrial stress initiate a shared downstream signaling pathway that represses global translation through phosphorylation of eIF2a [44]. The results demonstrated that the absence of TXNDC5 was unable to induce mitochondrial ROS in hepatocytes. Notwithstanding, these results diverge in the context of ER stress. All ER stressors reduced mitochondrial membrane potential in TXNDC5-KO cells. In WT cells, the presence of tunicamycin resulted in TXNDC5 maintaining mitochondrial stability, with no reduction in mitochondrial membrane potential observed (Figure 3). In this context, our previous findings indicate that TXNDC5 directly interacts with HSPA9 as a mitochondrial chaperone protein involved in protein folding and transport. The downregulation of HSPA9 in hepatocytes following the knockdown of TXNDC5 suggests that this protein may play a significant role in protecting mitochondria against tunicamycin [45].

**Figure 9:**
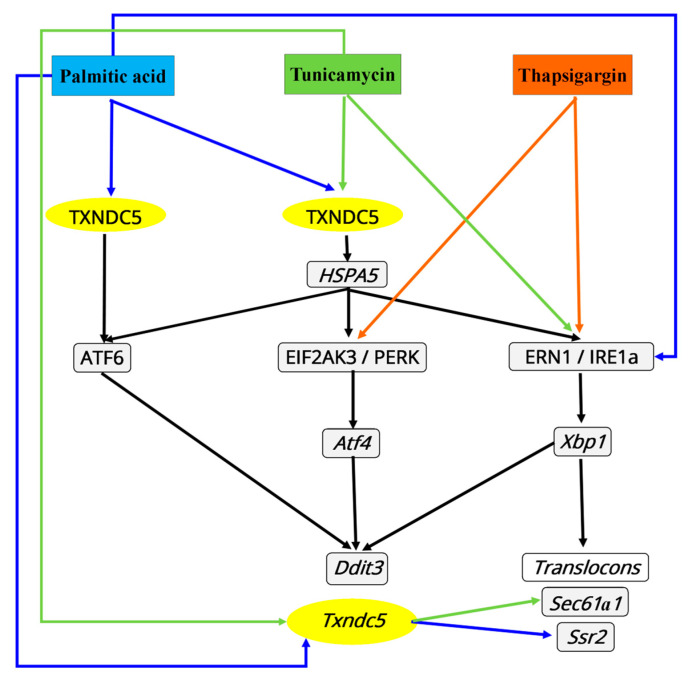
Putative involvement of TXNDC5 on the effect of ER stress triggers in the three different ER stress cascades.

The absence of TXNDC5 did not influence the expression of ATF6 in the presence of tunicamycin and thapsigargin (Figure 5A), but did suppress the induction raised by palmitic acid. Prior research has identified a unique positive feedback loop of the TGF1–ATF6–TXNDC5–TGFBR1 signaling axis in kidney, heart, and lung fibroblasts. This loop begins with TGF1, which causes the induction of TXNDC5 through ER stress and ATF6-mediated transcriptional control [46,47,48,49,50]. Our findings indicate that TXNDC5 may play a role in certain circumstances. Two ER stressors (tunicamycin and thapsigargin) do not appear to influence ATF6 levels, whereas palmitic acid appears to require TXNDC5 to induce this ATF6 effect (Figure 9).

The detachment of the chaperone HSPA5 from the luminal portion of the ER integral membrane proteins EIF2AK3, ERN1, and ATF6 is a crucial process in the activation of ER stress in human leukemia and bladder carcinoma cell lines [51]. Lee et al. demonstrated that the orphan nuclear receptor 4A1 (NR4A1) is tightly linked to *Txndc5*-induced transcriptional activity, and that defragmentation of the ER and altered expression of *Hspa5* are outcomes of *Txndc5* downregulation in pancreatic malignant cells [35]. The results obtained from the hepatic AML12 cell line indicate that the expression of HSPA5 is altered by the absence of TXNDC5 (Table 1). Furthermore, our results demonstrate that, in the absence of TXNDC5, ER stressors modify the protein level of HSPA5, with the exception of palmitic acid as a saturated fatty acid (Figure 5B). This observation provides evidence that HSPA5 may be involved in lipid metabolism, in addition to TXNDC5 [52]. In this context, HSPA5 exhibited elevated protein and mRNA levels in the group of rats fed a high-fat diet [53]. Furthermore, obese mice exhibited elevated hepatic levels of *Hspa5* mRNA in response to ATF6 and EIF2AK3 activation. In addition, *Hspa5* overexpression in hepatocytes reduces ER stress indicators, inhibits SREBP1c cleavage, and suppresses the transcription of SREBP1c and SREBP2 target genes, resulting in a significant reduction in hepatic cholesterol levels [54]. Furthermore, the activation of JNK as a central mediator of palmitic acid-induced hepatic lipoapoptosis causes the suppression of *Hspa5* in this cell line [55,56]. This saturated fatty acid has been demonstrated to induce lipotoxicity and insulin resistance in both mouse and human hepatocytes [32]. On the other side, our previous findings indicate that TXNDC5 is directly involved in the regulation of PRDX6 and the absence of TXNDC5 results in the downregulation of PRDX6 [45]. Therefore, the results of this study provide further evidence to support the link between TXNDC5 and the HSPA5 response to palmitic acid and it is possible that PRDX6 is involved in this pathway through a protein depalmitoylation [57]. 

**Figure 10:**
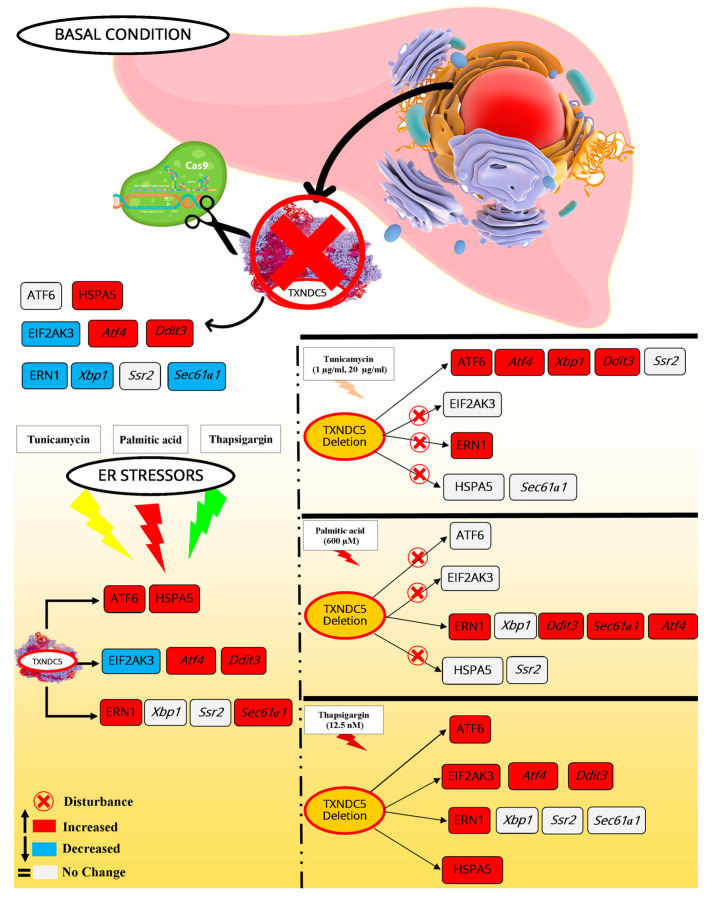
A summary of the findings of mRNA and protein expressions of ER stress sensors and their associated genes in response to different ER stressors, with and without TXNDC5. The scheme was created using Microsoft Publisher 2010. The symbols used to indicate the effects of ER stress on the expression of genes are as follows: red cross, disturbance in cascade; red color, increased expression; blue color, decreased expression; and white color, no changes.

The impact of TXNDC5 depletion on the EIF2AK3 cascade, previously designated as PERK, was also investigated. In the wild-type cell line, the mRNA expression levels of ER stress-related markers, including *Eif2ak3*, *Atf4*, and *Ddit3*, were found to be elevated in response to various ER stressors. The protein expression of EIF2AK3 exhibited a disparate pattern in tunicamycin and palmitic acid exposure, indicating that these stressors are involved in post-transcriptional regulation. However, TXNDC5-deficient AML12 hepatocytes exhibited a disruption in EIF2AK3 expression in the presence of tunicamycin and palmitic acid (Figure 6A). Negative feedback loops within the UPR result in a rapid downregulation of EIF2AK3-EIF2α signaling in CHO cells, due to the upregulation of *Ddit3* and its targets [58]. Furthermore, the reduction of EIF2AK3-EIF2α signaling has been observed to result in the development of diabetes phenotypes in mouse models of EIF2AK3 deficiency [59]. Conversely, the disruption of the EIF2AK3 pathway has been observed to result in the enhanced production of ROS [3] during ER stress in mouse fibroblasts [60]. The expression levels of *Eif2ak3* and *Txndc5* in NIH-3T3 fibroblasts exhibited a gradual increase over time under stressful conditions [48], suggesting a coordinated regulatory mechanism. On the other side, thapsigargin was found to promote the phosphorylation of EIF2AK3, which was markedly reduced by overexpression of *Hspa5* in hepatic steatosis in mice [39]. Thapsigargin was an exception in our experimental setting. In fact, the expression of EIF2AK3 in the presence of thapsigargin could be effectively stimulated in the absence of TXNDC5. Consequently, our findings indicate that TXNDC5 is essential for the induction of EIF2AK3 expression under specific stressful conditions in hepatocytes (Figure 10).

The absence of TXNDC5 had no effect on the expression of *Atf4* and *Ddit3* in AML12 cells under ER stress, as demonstrated in Figure 6B,C. Recent studies have demonstrated that the expression of *Atf4*, *Ddit3*, and apoptosis induced by NR4A1 (a modulator of TXNDC5) silencing regulates ER stress in MCF-7, RKO, MDA-MB-231, and Jurkat cell lines [35]. DDIT3 deletion has been observed to partially protect both cells and animals from ER stress-induced cell death, in contrast to overexpression of *Ddit3*, which has been shown to trigger cell death in the absence of other stimuli [61]. DDIT3 has been linked to the overexpression of death receptor 5 (DR5) and the downregulation of the anti-apoptotic protein BCL2 [62,63]. In contrast, DNA damage-inducible 34 protein (GADD34) has recently been identified in mouse fibroblasts as a target of DDIT3. Consequently, deletion of either DDIT3 or GADD34 protects cells against acute ER stress-induced cell death [64]. Consequently, the absence of TXNDC5 does not appear to suppress *Atf4* and *Ddit3* expression in AML12 cells under ER stress, suggesting that these cells may be induced to apoptosis. Our findings also indicate that TXNDC5 is not directly involved in the upregulation of *Atf4* and *Ddit3* expression in hepatocytes under ER stress conditions.

The reduction in one of the three ER stress markers frequently results in the activation of other ER stress markers in secretory goblet cells [65]. Both the EIF2AK3 and ERN1 pathways are implicated in the control of ER chaperones, ER-associated degradation, and other protective activities in mouse embryonic cells, NIH-3T3 fibroblasts, human embryonic kidney 293, and Phoenix-Eco cells [60,66,67,68,69]. Tsuchiya et al. also observed an increase in *Eif2ak3* expression in MIN6 cells lacking ERN1 protein [70]. Consequently, the disruption of EIF2AK3 in TXNDC5-deficient AML12 cells may result in increased ER stress and the activation of other ER stress sensors (Figure 10), such as ERN1. Indeed, the expression patterns of hepatocytes indicated that the expression of ERN1 could be increased in the context of ER stress when the expression of EIF2AK3 is disturbed in the absence of TXNDC5, as compared to the WT cells (Figure 6A and Figure 7A). It can be postulated that TXNDC5 plays a dynamic role in ER stress protection between the EIF2AK3 and ERN1 pathways in mouse hepatocytes, which is independent of the proapoptotic Bax (BCL2-associated X protein) and Bak (BCL2 antagonist of cell death) proteins, which have the potential to enhance ERN1 signaling [71,72]. This could explain why, despite the observed enhancement of ERN1 expression and EIF2AK3 disturbance, no changes in cell mortality were observed in TXNDC5-deficient hepatocytes.

X-box binding protein 1 (*Xbp1*) mRNA is the substrate of the endoribonuclease ERN1, which removes a 26-base intron [73]. It has been demonstrated that mice lacking ERN1 and XBP1 exhibit embryonic defects in liver formation and B lymphocyte differentiation [74,75]. The ERN1–XBP-1 signaling pathway has been linked to TXNDC5, as evidenced by the observation that the pharmacological inhibition of ERN1 and knockdown of XBP1 resulted in a reduction in TXNDC5 expression in pulmonary fibroblasts [48]. However, the present study in mouse hepatocytes indicates that the absence of TXNDC5 is associated with a reduction in *Xbp1* expression compared to the WT cells (Figure 7B). Moreover, it was observed that the absence or presence of TXNDC5 did not result in the induction of *Xbp1* by palmitic acid and thapsigargin.

Chen et al. proposed that ERN1 may regulate TXNDC5 through *Xbp1* mRNA splicing in lung fibroblasts [48] and that it plays a role in NAFLD, in which DDIT3, caspase-12, and JNK participate in ER stress by enhancing its activities [76]. In contrast, autophosphorylation of ERN1 may activate downstream genes without causing the *Xbp1* mRNA splicing, suggesting that ERN1 can exist in more states than just “on” and “off” in mouse embryonic fibroblasts and human pancreatic beta cells [77,78]. It has been postulated that XBP1 may interact with TXNDC5 indirectly [29], through binding to the promoter regions of downstream target genes, including HSPA5 in C2C12 myoblasts and MIN6 cells. This interaction may modulate *Txndc5* expression [79]. Our findings demonstrate that thapsigargin is an effective agent for converting *Xbp1* from its unspliced form to the spliced variant. Furthermore, the absence of TXNDC5 results in an increase in the latter form in AML12 cells (Figure 7C,D). In conclusion, the absence of TXNDC5 in hepatocytes modulates the splicing process of *Xbp1* in the context of thapsigargin exposure in the ERN1 cascade, which points to the role of TXNDC5 and its mRNA expression in this particular splicing process, and it is possible that ERN1 affects *Xbp1* splicing, not just its mRNA.

ERN1 and XBP1 target the translocon and translocon auxiliary components in mouse embryonic fibroblasts and HEK293T cells [66,80]. Studies in HeLa and Cos-7 cells have shown that XBP1 splicing is maximized when ERN1 binds to the translocons and SRP recruits unspliced XBP1 (XBP1u) to the ER [81,82]. This suggests that ERN1 may act in physical association with the translocons, unlike ATF6 or EIF2AK3 [82]. SEC61A1, which encodes an ER protein–translocation channel, and SSR2, a translocon auxiliary protein, are involved in SRP-mediated protein targeting to the ER [22]. Furthermore, repression of the SEC61A1 translocon subunits appeared to exclusively and specifically activate the ERN1 branch and also upregulated the SSR2 in human embryonic kidney cells and mouse embryonic fibroblasts [22,83]. The present report indicates that the *Ssr2* expression was not significantly induced by ER stress in TXNDC5 knockout cells (Figure 8A), although it was only induced by palmitic acid in WT cells. In contrast, the expression of *Sec61a1* was augmented in WT cells via all ER stressors. However, its expression exhibited no alterations in tunicamycin exposure in TXNDC5-KO cells (Figure 8B). This observation corroborates previous findings indicating that *Sec61a1* expression is uniquely regulated by the ERN1 cascade [80]. Moreover, translocons may also be regulated by other mediators, such as DDIT3 and HSPA5. The suppression of SEC61A1 and SEC61B has been observed to stimulate *Ddit3* expression in myelogenous leukemia cells [84]. However, DDIT3 was upregulated in SEC61A1-induced HeLa cells [85], which is consistent with our findings. In addition, *Txndc5*, *Sec61a1*, and *Ssr2* are upregulated in XBP-1-transduced NIH-3T3 fibroblasts and murine macrophage cells [86,87]. However, another study indicated that in the presence of sertraline as an anti-stress drug in HepG2 cells, the mRNA level of *Txndc5* decreased and *Ssr2* and *Ern1* increased [88]. Consequently, it is plausible that the regulation of translocons by DDIT3 and HSPA5 is contingent upon the specific tissue in question. These results indicate a feedback paradigm where TXNDC5 can regulate translocon expression by modulating the expression of the ERN1 cascade via the XBP1 mediator in ER stress conditions. Additionally, ERN1 may be utilized to monitor the status of the translocon in mouse hepatocytes. Furthermore, the expression of *Ssr2* and *Sec61α1* ER protein–translocon channels is selectively influenced by the absence of TXNDC5, with the influence dependent on the source of ER stress.

## 4. Materials and Methods

### 4.1. Generation of TXNDC5 Knockout AML12 Cells

The Alpha mouse liver (AML12) cell line with a stable knockout of TXNDC5 was generated as previously described [28]. The cells were transfected with TXNDC5/ERp46 HDR and TXNDC5 CRISPR/Cas9 KO plasmids (Santa Cruz Biotechnology, Dallas, TX, USA) using lipofectamine 3000 (Thermo Fisher Scientific, Waltham, MA, USA). The TXNDC5 CRISPR/Cas9 KO plasmid contains a gRNA sequence, 5′-TTATCAAGTTCTTCGCTCCG-3′, which generates a double-stranded break (DSB) specifically in the fifth exon of the *Txndc5* gene. After multiple rounds of puromycin incubation, puromycin-resistant AML12 TXNDC5-KO cells were selected. RNA and Western blot analyses confirmed the deletion of TXNDC5 (Supplementary Figure S1).

### 4.2. Cell Culture and Treatment

The AML12 cell line (WT) was obtained from the ATCC collection (Manassas, VA, USA) and generated TXNDC5-KO AML12 cells (KO) cultured in a 6-well plate (in duplicate) at 37 °C in a humidified atmosphere of 5% CO_2_ in Dulbecco’s modified Eagle’s minimum essential medium (DMEM; Thermo Fisher Scientific, Waltham, MA, USA): F-12-Ham’s medium (GE Healthcare Life Science, South Logan, UT, USA) at a 1:1 ratio supplemented with 10% fetal bovine serum (Thermo Fisher Scientific, Waltham, MA, USA), 1:500 insulin-transferrin-selenium (Corning, Bedford, MA, USA), 40 ng/mL dexamethasone (Sigma-Aldrich; Merck Millipore, Darmstadt, Germany), 1% nonessential amino acids (Thermo Fisher Scientific, Waltham, MA, USA), 1% amphotericin B (1000 mg/mL; Thermo Fisher Scientific, Waltham, MA, USA), 1% penicillin (1000 U/mL; Thermo Fisher Scientific), and 1% streptomycin (1000 mg/mL; Thermo Fisher Scientific, Waltham, MA, USA). After reaching 90–100% confluence, the AML12 cells were given fresh medium without fetal bovine serum and amphotericin B. For RNA isolation, the cells were treated with 1 and 20 µg/mL of tunicamycin (Sigma-Aldrich, Merck Millipore, Darmstadt, Germany), 12.5 nM of thapsigargin (Sigma-Aldrich, Merck Millipore, Darmstadt, Germany), and 600 µM of palmitic acid (Sigma-Aldrich, Merck Millipore, Darmstadt, Germany) for 24 h.

### 4.3. MTT Assay

Cell viability was assessed using the 3-(4,5-dimethylthiazol-2-yl)-2,5-diphenyltetrazolium bromide test (MTT; Sigma-Aldrich, Merck Millipore, Darmstadt, Germany). The cells were seeded at a density of 5000 cells/well on a 96-well plate and exposed to a range of 1 to 20 nM of thapsigargin (Sigma-Aldrich, Merck Millipore, Darmstadt, Germany) for 24 h. Tunicamycin (Sigma-Aldrich, Merck Millipore, Darmstadt, Germany) was dissolved in 0.1% DMSO to a concentration of 1 to 20 µg/mL, while palmitic acid (Sigma-Aldrich, Merck Millipore, Darmstadt, Germany) was dissolved in ethanol to a concentration of 50 to 4000 µM. The culture medium was then supplemented with 1 mg/mL of MTT. After a 3-h incubation, the cell growth medium was replaced with DMSO and the absorbance was measured at 570 nm using a SPECTROstar^®^ Nano Microplate Reader (Omega, BMG Labtech, Ortenberg, Germany). The IC50 survival rates of AML12 WT and TXNDC5-KO cells in ER stress circumstances were obtained using this method.

### 4.4. TXNDC5 Promoter Plasmid Construction and Transfection

To investigate the activity of the *Txndc5* promoter, we constructed expression plasmids by inserting PCR-amplified 2024 bp length *Txndc5* promoter (nucleotides 38,527,915 to 38,529,935 of GRCm38.p6 *Mus musculus* assembly) into the EcoRI site of the pEZX-GA01 expression vector (GeneCopoeia, Rockville, MD, USA) following the CloneAmp™ HiFi PCR Premix and In-Fusion^®^ HD Cloning Kit Protocols (Takara Bio, San Jose, CA, USA). AML12 cells were plated at a density of 1 × 10^4^ cells/well in 96-well plates containing Dulbecco’s modified Eagle’s medium supplemented with 10% fetal bovine serum. After 24 h, LipofectAMINE 3000 reagent (Invitrogen, Carlsbad, CA, USA) was used to transfect the cells with 100 ng of the constructed plasmid, following the manufacturer’s protocol. The cells were then treated with thapsigargin (6.25 nM), palmitic acid (300 µM), and tunicamycin (20 µg/mL) for 24 h. 

### 4.5. Alkaline Phosphatase Assay

The transfected cell culture medium was collected and heated at 65 °C for 15 min. Then, 100 µL of 4-nitrophenyl phosphate disodium salt hexahydrate (1 mg/mL) dissolved in deionized water (Sigma-Aldrich, Merck Millipore, Darmstadt, Germany) was added to 10 µL of each sample in a microplate. Absorbance was measured at a wavelength of 405 nm using a SPECTROstar^®^ Nano microplate reader (Omega, BMG Labtech, Ortenberg, Germany).

### 4.6. Luciferase Activity Assay

The luciferase assay was conducted based on the ratio of luciferase to alkaline phosphatase units. Coelenterazine (6 µM) dissolved in deionized water (Sigma-Aldrich, Merck Millipore, Darmstadt, Germany) was added to 10 µL of culture medium of transfected cells in a microplate. The intensity of luminescence was measured with a microplate reader (FLUOstar^®^, Omega, BMG Labtech, Ortenberg, Germany) using an emission filter with a gain of 3600.

### 4.7. RNA Extraction

Total cellular RNA was extracted using a Quick-RNA^TM^ MiniPrep kit (Zymo Research, Tustin, CA, USA) following the manufacturer’s instructions. RNA concentration was measured at 260/280 nm wavelengths using a SPECTROstar^®^ Nano microplate reader (Omega, BMG Labtech, Ortenberg, Germany). The integrity of the 28S and 18S ribosomal RNAs was confirmed by electrophoresis on a 1% agarose gel followed by ethidium bromide staining (Sigma-Aldrich, Merck Millipore, Darmstadt, Germany), and the 28S/18S ratio was greater than 2.

### 4.8. Quantitation of mRNA by RT-qPCR

To measure gene RNA expression, cDNA was prepared from 500 ng of total RNA using the PrimeScript RT reagent kit (TaKaRa Biotechnology, Kusatsu, Shiga, Japan) following the manufacturer’s instructions. The cDNA was then analyzed using a Step One Plus Real-Time PCR System (Applied Biosystems, Foster City, CA, USA) with SYBR Green PCR Master Mix (Applied Biosystems, Foster City, CA, USA) according to the manufacturer’s guidelines. The primers for RT-qPCR were designed using Primer Express (Applied Biosystems, Foster City, CA, USA). They were then validated for gene specificity and amplification of cDNA rather than genomic DNA using BLAST analysis (NCBI). Finally, the primers were selected based on their efficiency (Table S1). The comparative 2^−ΔΔCT^ method was used to calculate the relative ratio of each gene’s transcript expression level to the mean values of control samples. The results were normalized to the reference genes *Ppib* and *Tbp*.

### 4.9. Estimation of Xbp1 mRNA Splicing

To estimate the extent of *Xbp1* mRNA splicing, 500 ng of cDNA was amplified with specific primers using CloneAmp™ HiFi PCR Premix (TaKaRa Biotechnology, Kusatsu, Shiga, Japan) in a standard thermocycler. The primers used were Mouse *Xbp1* forward, 5′-GAG AAC CAG GAG TTA AGA ACA CG-3′ and reverse, 5′-GAA GAT GTT CTG GGG AGG TGA C-3′ [70]. To evaluate the spliced and unspliced forms of *Xbp1* mRNA, the PCR products were separated by electrophoresis on 4% agarose gels and visualized by ethidium bromide staining (Sigma-Aldrich, Merck Millipore, Darmstadt, Germany). The intensities of the bands were then measured to determine the extent of *Xbp1* splicing by Quantity One software version 4.6.8 (Bio-Rad, Hercules, CA, USA).

### 4.10. Western Blot

Proteins were extracted from AML12 WT and TXNDC5-KO cells that were treated with stressors, as explained above. The proteins were quantified and transferred to a polyvinylidene difluoride (PVDF) membrane (Bio-Rad, Hercules, CA, USA)., following the methodology described in previous publications [28,89]. The membranes were then blocked with phosphate-buffered saline (PBS) containing 5% bovine serum albumin (BSA) for 1 h at room temperature. Following blocking, the membranes were incubated overnight at 4 °C with rabbit primary polyclonal antibody against mouse TXNDC5 (1:1000, Proteintech, Manchester, UK), mouse monoclonal anti-HSPA5 (1:1000, Proteintech, Manchester, UK), mouse monoclonal anti-ATF6 (1:500, Proteintech, Manchester, UK), rabbit monoclonal anti-EIF2AK3 (1:500, Cell Signaling Technology, Danvers, MA, USA), rabbit monoclonal anti-ERN1 (1:500, Thermo Fisher Scientific, Waltham, MA, USA), rabbit monoclonal anti-β-ACTIN (1:2000, Sigma, St. Louis, MO, USA), and mouse monoclonal anti-β-ACTIN (1:2500, Sigma, St. Louis, MO, USA). The membranes were washed with a PBS solution containing 0.1% Tween 20 before incubation for 1 h at room temperature with a conjugated goat anti-rabbit IgG (H&L) DyLight 800 secondary antibody (1:15,000, Thermo-Scientific, Waltham, MA, USA) or a goat anti-mouse IgG (H&L) DyLight 680 secondary antibody (1:30,000, Thermo-Scientific, Waltham, MA, USA). The blots were captured using the Odyssey^®^ Clx (LI-COR, Bad Hamburg, Germany). The blots were quantified using Image Studio Lite Version 5.2 software from LI-COR Biosciences GmbH in Bad Homburg, Germany. The densitometric values were normalized to the housekeeping antibodies and expressed in arbitrary units.

### 4.11. ROS Assessment with Flow Cytometry

To quantify cellular ROS, flow cytometry was used to assess AML12 WT and TXNDC5-KO cell lines. The cells were treated with 10 µL of 2.0 mg/mL 2,7-dichlorofluorescein diacetate (DCFH-DA; Sigma-Aldrich, Merck Millipore, Darmstadt, Germany) dissolved in fresh PBS for 30 min at 37 °C. After exposure, the cells were trypsinized, washed, collected, centrifuged, and resuspended to 7.5 × 10^5^ cells/mL in PBS. The fluorescence within the cells was measured using flow cytometry (Beckman Coulter, Brea, CA, USA) with excitation/emission spectra of 485/520 nm, respectively. A total of 30,000 cells were analyzed for all conditions, and the data were processed using Kaluza Analysis Version 1.5a software.

### 4.12. Intracellular ROS Production

AML12 WT and TXNDC5-KO cells were seeded at a density of 5000 cells per well in a 96-well plate and cultured for 72 h at 37 °C. The cells were treated with thapsigargin, palmitic acid, and two different concentrations of tunicamycin for 24 h. The treatments were administered in medium free of fetal bovine serum and amphotericin B at a concentration of 6.25 nM, 300 µM, 1 and 20 µg/mL of the respective substances. Subsequently, 10 µL of 2.0 mg/mL DCFH-DA (Sigma-Aldrich, Merck Millipore, Darmstadt, Germany) dissolved in fresh PBS was added to the cells. After 30 min at 37 °C, the medium was removed, and the presence of reactive oxygen species was assessed by measuring the conversion of DCFH-DA into fluorescent DCF using a microplate reader (FLUOstar^®^, Omega, BMG Labtech, Ortenberg, Germany) at excitation and emission wavelengths of 485 and 520 nm, respectively.

### 4.13. Mitochondrial Membrane Potential Assay

The mitochondrial membrane potential assay (Ab228569 TMRM assay kit, Abcam, Waltham, MA, USA) was used to determine potential mitochondrial damage caused by different ER stressors in AML12 WT and TXNDC5-KO cells. The cells were seeded in 96-well plates at a concentration of 5 × 10^5^ cells/mL and exposed to thapsigargin, palmitic acid, and tunicamycin at concentrations of 6.25 nM, 300 µM, and 1 and 20 μg/mL, respectively, for 24 h. After exposure, the cells were washed with PBS and 1µM TMRM was added. After 30 min incubation in 37 °C, the cells were washed twice with PBS/0.2% BSA. Then, 1X live cell imaging buffer was added. The fluorescence intensity was measured using a microplate reader (FLUOstar^®^, Omega, BMG Labtech, Ortenberg, Germany) with an excitation/emission of 548/575 nm. 

### 4.14. TMRM Microscopy Procedure

To confirm the TMRM results, the microscopy procedure was also performed with 200 nM of TMRM (Ab228569 TMRM assay kit, Abcam, Waltham, MA, USA). Additionally, we used 20 µM carbonylcyanide 4-(trifluoromethoxy)phenylhydrazone (FCCP) as a positive control for 10 min prior to staining with TMRM. The cell’s nucleus was stained using NucBlue Live Ready Probes Reagent (Invitrogen, Thermo-Scientific, Waltham, MA, USA) and incubated for 20 min at 25 °C. The cells were evaluated using a FLoid Cell Imaging Station (Life Technologies, Invitrogen, Thermo-Scientific, Waltham, MA, USA) that was equipped with bandpass filters capable of visualizing EX/EM = 548/573. The data were analyzed using ImageJ (Fiji) software version windows 64-bit Java 8.

### 4.15. Statistical Analysis

The figures display means ± standard deviations. Statistical significance was calculated using GraphPad Prism 8 for Windows (GraphPad, San Diego, CA, USA). The normal distribution of the data and homogeneity of variance among groups were assessed using the Shapiro–Wilk test and Bartlett’s or Levene’s tests, respectively. Statistical differences were calculated using the one-tailed Mann–Whitney’s U- or Student’s *t*-tests. The figure legends indicate the *p*-values, denoted as *, *p* < 0.05; **, *p* < 0.01; ***, *p* < 0.001, and ****, *p* < 0.0001.

## 5. Conclusions

The results of the current study indicate that TXNDC5 deficiency is associated with a reduction in mRNA expression of *Ern1*, *Xbp1*, *Eif2ak3*, and *Sec61a1* and an increase in those of *Atf4*, *Hspa5*, and *Ddit3* in mouse hepatocytes. The expression of *Atf6* and *Ssr2* was not significantly altered in the absence of TXNDC5. The protein expression results of prominent ER stress markers indicated that the ERN1 and EIF2AK3 proteins were downregulated, while the HSPA5 protein was upregulated. Moreover, the ATF6 protein did not exhibit any significant changes in the absence of TXNDC5 at the protein level. The knockout of TXNDC5 has been demonstrated to increase cellular ROS production in AML12 cells. Furthermore, TXNDC5 activity is required to maintain normal mitochondrial function during tunicamycin-induced ER stress. The enhancement of TXNDC5 in AML12 cells is dependent on the induction of ER stress by thapsigargin, palmitic acid, and tunicamycin. Tunicamycin, an inhibitor of glycosylation, has been observed to disrupt the protein levels of HSPA5, ERN1, and EIF2AK3 in TXNDC5-deficient cells. Additionally, tunicamycin has been shown to affect the mRNA levels of *Eif2ak3* and *Sec61a1* in TXNDC5-deficient cells. However, palmitic acid, which is an inducer of excessive saturated fatty acids, can disarrange ATF6, HSPA5, and EIF2AK3 at the protein level and *Atf6*, *Hspa5*, *Eif2ak3*, and *Ssr2* at the gene expression level in TXNDC5 knockout cells. The absence of TXNDC5 did not affect any ER stress cascades in thapsigargin-induced ER stress in hepatocytes, despite the activation of the ERN1–XBP1 pathway under these conditions. Furthermore, the absence of TXNDC5 can induce the Xbp1 spliced form. Moreover, it is conceivable that the upregulation of ERN1 may alter the splicing pattern of Xbp1, rather than merely influencing its mRNA expression. In summary, TXNDC5 can selectively activate different ER stress cascades via HSPA5, depending on the source of ER stress. Conversely, the absence of TXNDC5 can disrupt the EIF2AK3 cascade.

### Limitations and Future Research

To address the potential limitations of cell viability as an endpoint, we plan to include a broader range of functional assays in future experiments. These could include assessments of lipid accumulation, inflammatory cytokine production, and markers of liver injury and fibrosis. Such approaches would provide a more comprehensive understanding of the impact of TXDNC5 on liver health under stress conditions. In addition, it is important to consider the long-term effects of TXDNC5 deficiency. Chronic stress conditions or prolonged exposure to ER stress inducers may reveal cumulative effects on cell viability and function that are not apparent in short-term assays. Finally, future studies could examine the effects of TXDNC5 KO on the expression of proteins involved in lipid metabolism, inflammation, and fibrosis, which are key components of NAFLD progression.

## Figures and Tables

**Table 1:** The mRNA and protein expression of ER indicators in the absence of TXNDC5 in basal conditions.

Gene or Protein Symbol	mRNA Expression (Fold Change)		Protein Expression (Arbitrary Units)		Changes
	Wild-Type	TXNDC5-KO	Wild-Type	TXNDC5-KO	KO/WT
ATF6	1.0 ± 0.1	1.1 ± 0.2	105 ± 17	87 ± 36	No change
ERN1	1.0 ± 0.1	0.7 ± 0.1	100 ± 11	41 ± 20	Downregulation *
EIF2AK3	1.0 ± 0.1	0.8 ± 0.1	100 ± 9	18 ± 11	Downregulation *
HSPA5	1.0 ± 0.1	1.3 ± 0.2	100 ± 7	131 ± 34	Upregulation *
*Xbp1*	1.0 ± 0.2	0.4 ± 0.2	NA	NA	Downregulation **
*Sec61α1*	1.0 ± 0.1	0.7 ± 0.1	NA	NA	Downregulation **
*Atf4*	1.0 ± 0.2	1.4 ± 0.3	NA	NA	Upregulation **
*Ddit3*	1.0 ± 0.2	2.4 ± 0.5	NA	NA	Upregulation **
*Ssr2*	1.0 ± 0.1	0.9 ± 0.1	NA	NA	No change

Data are means and standard deviations. The Mann–Whitney’s U-tests were used in the data analyses. * *p* < 0.05, ** *p* < 0.01.

## Data Availability

Data are contained within the article and Supplementary Material.

## Supplementary Materials

The following supporting information can be downloaded at: https://www.mdpi.com/article/10.3390/ijms25137128/s1.

## Abbreviations

NAFLD	Non-alcoholic fatty liver disease
NASH	Non-alcoholic steatohepatitis
ATF6	Activating transcription factor 6
HSPA5	Heat shock protein 5
EIF2AK3	Eukaryotic translation initiation factor 2 alpha kinase 3
ATF4	Activating transcription factor 4
DDIT3	DNA-damage inducible transcript 3
ERN1	Endoplasmic reticulum (ER) to nucleus signaling 1
XBP1	X-box binding protein 1
SSR2	Signal sequence receptor, beta
SEC61A1	Sec61 alpha 1 subunit
*Tbp*	TATA-box binding protein
*Ppib*	Peptidylprolyl isomerase B
TXNDC5	Thioredoxin domain containing 5
WT	Wild-type AML12
KO	TXNDC5-Knockout AML12
ER	Endoplasmic reticulum
CRISPR	Clustered regularly interspaced short palindromic repeats
UPR	Unfolded protein response
PDI	Protein disulfide isomerase
MTT	3-(4 5-dimethylthiazol-2-yl)-2 5-diphenyltetrazolium bromide
ROS	Reactive oxygen species
AML12	Alpha mouse liver cell line
NIH-3T3	Mouse NIH/Swiss embryo fibroblasts
MCF-7	Michigan Cancer Foundation-7
RKO	Human colon carcinoma cell line
MDA-MB-231	M.D. Anderson-metastatic breast 231
Jurkat	Immortalized line of human T lymphocyte cells
L3.6pL	L3.6 pancreas-liver cell line
MIN6	Mouse insulinoma cell line 6
C2C12	C2C12 mouse myoblast cell line
HEK293T	Human embryonic kidney cells 293T
HeLa	Henrietta Lacks cancer cells
COS-7	Monkey African green kidney fibroblasts
