# Supplementary material for: TXNDC5 Plays a Crucial Role in Regulating Endoplasmic Reticulum Activity through Different ER Stress Signaling Pathways in Hepatic Cells
Source: Int J Mol Sci. 2024 Jun 28;25(13):7128. doi: 10.3390/ijms25137128 (PMC11241358; doi:10.3390/ijms25137128)
Supplement: Supplementary file 1 [file ijms-25-07128-s001.zip › ijms-3060485-supplementary.pdf]

Supplementary Table S1. Sequences of real-time PCR primers according to MIQE guidelines

Gene symbol	Primer sequence, sense/antisense (5'->3')	Amplicon length	Accession	Exon	Biological Process	Concentration	Efficiency
Atf6	AGAGTCTGCTTGTCAGTCGC<br>GGTTCTCTGACACCACCTCG	149	NM_001081304.1 ,<br>XM_030253420.2,<br>XM_011238796.4,<br>XM_006496792.5	8/9	Unfolded protein response (UPR) during ER stress	0.2 µM	99%
Hspa5	CTTGCCATTCAAGGTGGTTG<br>TGCATGGGTAACCTTCTTTCCC	163	NM_022310.3,<br>NM_001163434.1	3/5	Folding and assembly of proteins in the ER and ER homeostasis	0.2 µM	98%
Eif2ak3	TATGTTGGAAGGCTTGAGGTCC<br>GTACATTCAGATGCAGCTGTGC	178	NM_010121.3,<br>XM_011241202.3,<br>XM_006505501.2	13/<br>14	Unfolded protein response (UPR) and integrated stress response (ISR) during ER stress	0.2 µM	96%
Atf4	CAGCAGTGTTGCTGTAACGG<br>ATCTCGGTCATGTTGTGGGG	85	NM_009716.3,<br>NM_001287180.1	2/3	Integrated stress response (ISR)	0.2 µM	97%
Ddit3	GCGACAGAGCCAGAATAACA<br>GATGCACTTCCTTCTGGAACA	168	NM_007837.4,<br>XM_006513197.4	1/2	Adipogenesis and erythropoiesis in ER stress response	0.2 µM	95%
Ern1	AACAACCTGCCCAAACATCG<br>TGGTCGGTGTGTTGTCTGAA	109	NM_023913.2	11/<br>12	Unfolded protein response (UPR) during ER stress	0.2 µM	93%
Xbp1	GAGAACCAGGAGTTAAGAACACG<br>GAAGATGTTCTGGGGAGGTGAC	157 &<br>183	NM_001271730.1,<br>NM_013842.3	3/5	Unfolded protein response (UPR) during ER stress	0.4 µM	94%
Ssr2	TTGGCTCTGTTAGCCGTCAG<br>TTGAGAGGACGCAGGACAAC	272	NM_001356316.1,<br>NM_001356317.1,<br>NM_025448.4,<br>NM_001356319.1,<br>NM_001356318.1	2/4	Protein translocation across the ER membrane	0.2 µM	96%
Sec61a1	TCTGCAAAAAGGGTACGGCT<br>GTTCTGGCGGTAGAATGCCT	211	NM_016906.4	7/8	Transport of signal peptide-containing precursor polypeptides across the ER	0.2 µM	99%
Tbp	GTGAGTTGCTTGCTCTGTGC<br>GCTGCGTTTTTGTGCAGAGT	359	NM_013684.3	8	Housekeeping gene	0.2 µM	98%
Ppib	GGAGATGGCACAGGAGGAA<br>TAGTGCTTCAGCTTGAAGTTCTCAT	71	NM_011149.2	3/4	Housekeeping gene	0.2 µM	99%
Txndc5	CAGGCTTGTCAGATGTCACCAT<br>TAACCTCGTACCGAGTACTTGCTG	82	NM_001289599.1,<br>NM_001289598.1,<br>NM_145367.4	9/1<br>0	Formation of disulfide bonds in proteins	0.2 µM	92%

Abbreviations: *Atf6*, activating transcription factor 6; *Hspa5*, heat shock protein 5; *Eif2ak3*, eukaryotic translation initiation factor 2 alpha kinase 3; *Atf4*, activating transcription factor 4; *Ddit3*, DNA-damage inducible transcript 3; *Ern1*, endoplasmic reticulum (ER) to nucleus signalling 1; *Xbp1*, X-box binding protein 1; *Ssr2*, signal sequence receptor, beta; *Sec61a1*, Sec61 alpha 1 subunit; *Tbp*, TATA-box binding protein; *Ppib*, peptidylprolyl isomerase B; *Txndc5*, thioredoxin domain containing 5

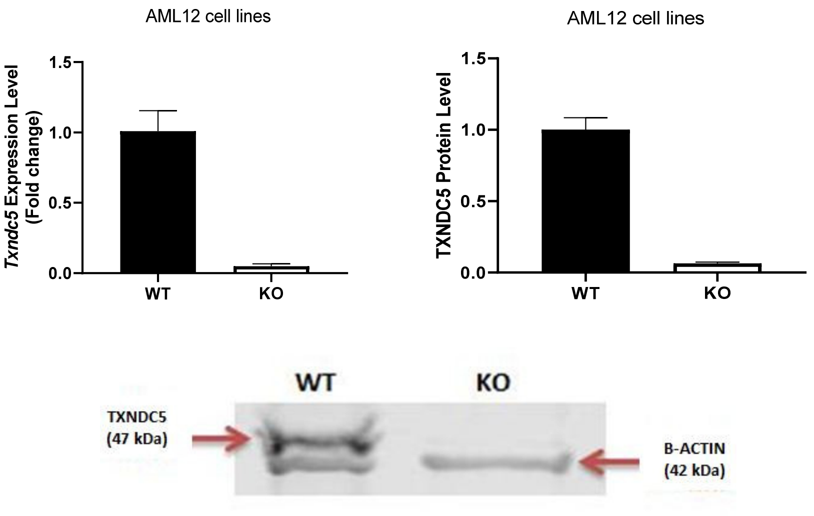

Supplementary Figure S1. Characterization of AML12 cell lines. *Txndc5* mRNA and protein levels in normal mouse hepatocyte AML12 cells (wildtype (WT)) and TXNDC5-deficient AML12 cells (knockout (KO))

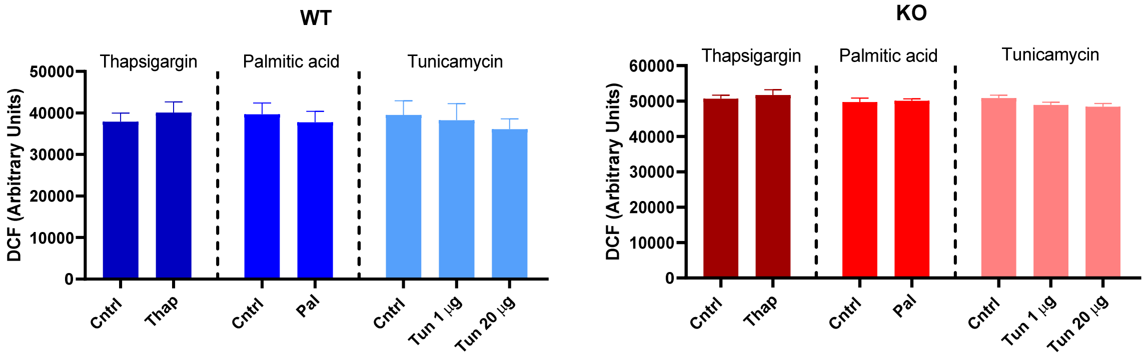

Supplementary Figure S2. ROS assessment of WT and KO AML12 cell lines under thapsigargin, palmitic acid, and tunicamycin stress.
